# Supplementary material for: Promoter-proximal elongation regulates transcription in archaea
Source: Nat Commun. 2021 Sep 17;12:5524. doi: 10.1038/s41467-021-25669-2 (PMC8448881; doi:10.1038/s41467-021-25669-2)
Supplement: Supplementary file 1 — Supplementary Information [file 41467_2021_25669_MOESM1_ESM.pdf]

Supplementary Information:

Promoter-proximal elongation regulates transcription in archaea

Fabian Blombach, Thomas Fouqueau, Dorota Matelska, Katherine Smollett, Finn Werner

Supplementary Table 1: List of primers used to generate in vitro transcription templates and 25 nt antisense oligonucleotides for the affinity purification*

PROMOTER	PRIMER NAME	SEQUENCE
thsB	thsB fw	5′ CGTATTTGATTTGAGTAAAATTTTT 3′
	thsB rv	5′ TCTTTTCCATATGTTCTACTTGA 3′
	thsB antisense	5′ GCTGTTGCCATTTTACGCACCTTCC 3′
rps8e	rps8e fw	5′ GTAACTTTGGGGCCTAA 3′
	rps8e rv	5′ ATTTCCAATTTCATATTTTCTCT 3′
	rps8e antisense	5′ TTGGTAAAATCCCATAATTTACGCCTCT 3′
dhg-1	dhg-1 fw	5′ AGTTGTATTGAGCATAAAATTTAT 3′
	dhg-1 rv	5′ TGTTCTTATCTTTATTTTCCCGT 3′
	dhg-1 antisense	5′ GGGTTTCACTATTATAGCTTTCATT 3′
CRISPR C	crispr C fw	5′ AACGCAAACCAGAGAAAAGCT 3′
	crispr C rv	5′ TATAAGAGATTATCTTAGCCCAGATGAAGCAA 3′
	crispr C antisense	5′ TCTGAGGGTTTATTAATCTCTTACT 3′

* For *rps8e* a 28 nt oligonucleotides was used for the affinity purification because transcription initiation appeared to occur at a 4 bp cluster of TSSs.

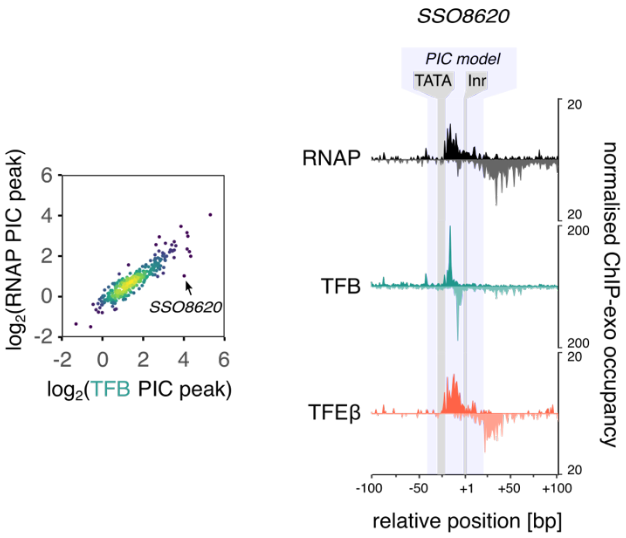

Supplementary Figure 1: Lower RNAP recruitment is associated with narrower TFB ChIP-exo footprints

ChIP-exo profile of the *SSO8620* promoter that shows low RNAP and TFEβ ChIP-exo signal relative to TFB. The left panels show the quantifications for RNAP and TFB PIC signal as in Figure 1d with the position of *SSO8620* indicated. The right panel shows the ChIP-exo signal for RNAP, TFB, and TFEβ. RNAP and TFEβ profiles show similar broad footprints as for the aggregate profiles in Figure 1b that can be attributed to PICs. In contrast, the TFB profile is dominated by a narrower footprint that can be attributed to ternary TFB-TBP-DNA complexes lacking RNAP and TFEβ. All ChIP-exo data represent the geometrical mean of three biological replicates.

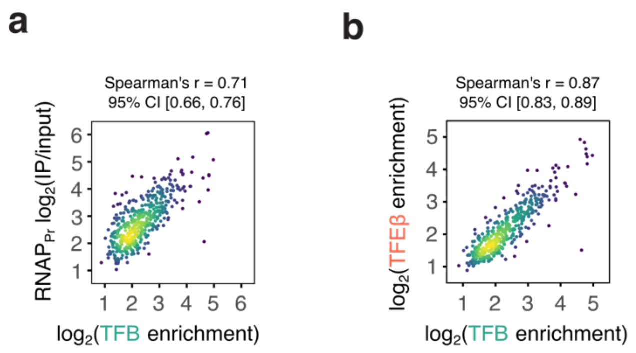

Supplementary Figure 2: ChIP-seq data are consistent with uniform PIC assembly during exponential growth

(a) and (b) Scatter plots depicting correlation of TFB promoter occupancy with RNAP (a) and TFEβ promoter occupancy (b) under exponential growth conditions based on ChIP-seq data. 454 promoters with unambiguous assignment of TFB and TFEβ peaks to mapped TSSs or start codons (if mapped TSS was not available) and 100 bp minimal TU length were included. Data show the geometric mean of two biological replicates. These data are overall consistent with ChIP-exo data (Figure 1de). Because of the limited resolution of ChIP-seq occupancy data, the RNAP promoter occupancy quantification ($RNAP_{Pr}$) comprises RNAP present in PICs as well as promoter-proximal TECs. Therefore, the correlation of $RNAP_{Pr}$ with with TFB is lower than the corresponding ChIP-exo signal correlation (Figure 1d) or the correlation between TFB and TFEβ which are both specific to PICs (panel b).

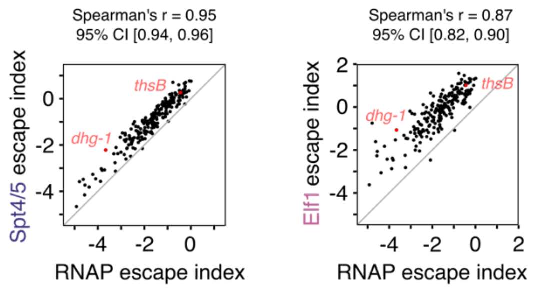

Supplementary Figure 3: Correlated promoter-proximal accumulation of elongation factors and RNAP

Scatter plots depicting the correlation between escape indices calculated for RNAP and elongation factors Spt4/5 (left panel) and Elf1 (right panel) for 212 TUs. Escape indices represent the mean of two biological replicates.

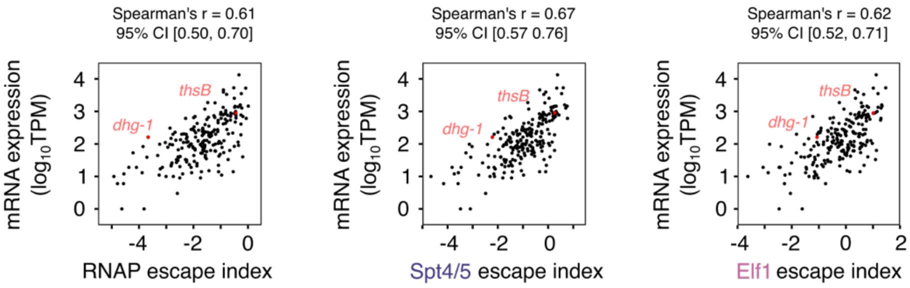

Supplementary Figure 4: TEC escape correlates with higher mRNA expression

Scatter plots depicting the correlation between mRNA expression levels and the escape indices calculated for RNAP and elongation factors Spt4/5 (left panel) and Elf1 (right panel) for 211 protein-encoding TUs. Escape indices and mRNA expression values represent the mean of two biological replicates.

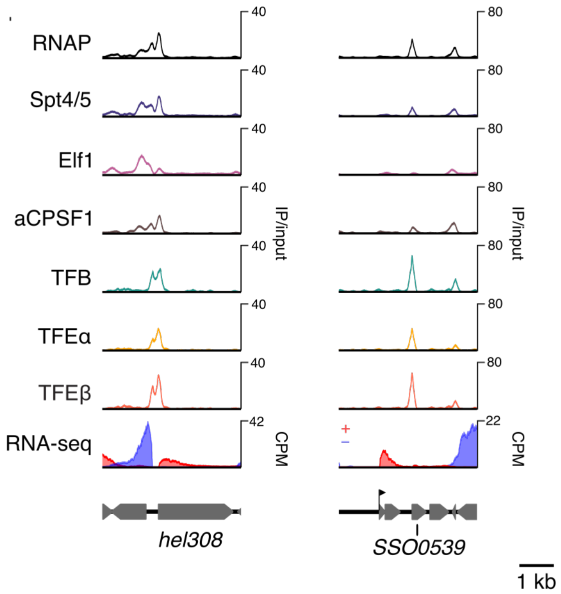

Supplementary Figure 5: Examples of low escape TUs showing poor Elf1 and aCPSF1 recruitment

ChIP-seq occupancy plots of two exemplary TUs: *hel308* coding for a DNA helicase and *SSO0539* coding for a hypothetical protein. Traces show mean occupancy for two biological replicates with the range depicted as semi-transparent ribbon.

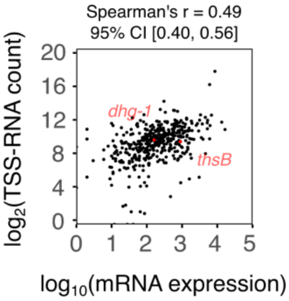

Supplementary Figure 6: Correlation between TSS-RNAs and mRNA levels

Scatter plot depicting the correlation between mRNA expression levels and TSS-RNA (n=438 TUs). TSS-RNA data represent the geometric mean of two biological replicates. Rockhopper [1] estimates of mRNA levels are based on two biological replicates.

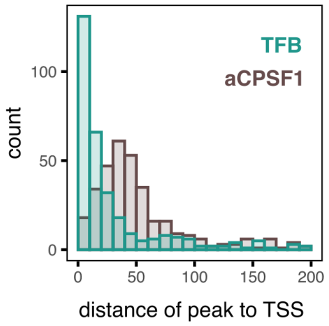

Supplementary Figure 7: aCPSF1 occupancy increases in the promoter proximal region.

To study aCPSF1 occupancy in promoter regions isolated from transcription termination sites, we calculated the distance between aCPSF1 peaks (n = 1633) and mapped TSSs or start codons of genes in divergent orientation with maximum size of the intergenic region of <500 bp (n= 954). Initiation factor TFB peaks (n = 1657) were analysed likewise as control. The histogram shows counts for peaks within <200 bp distance to the putative start sites, n = 311 and n = 331 for TFB and aCPSF1, respectively. Binwidth = 10 bp.

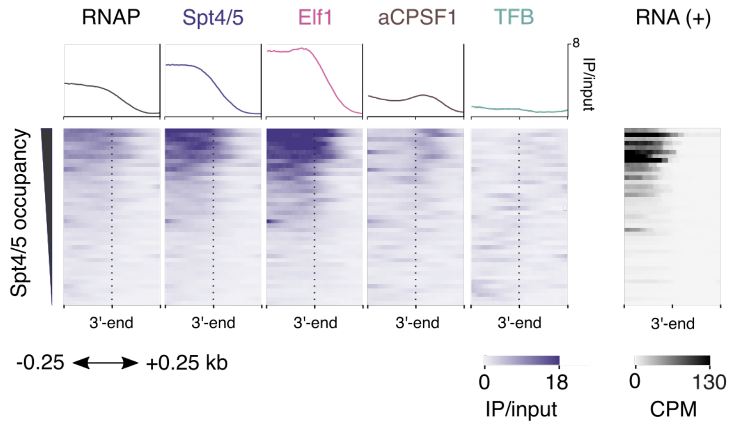

Supplementary Figure 8: aCPSF1 association with transcription termination sites. Heatmap of RNAP, elongation factors Spt4/5 and Elf1 and termination factor aCPSF1 ChIP-seq occupancy at 41 predicted RNA 3′-ends during exponential growth. Data represent one representative of two biological replicates. RNA 3′-ends as putative termination sites were predicted based on RNA-seq data and filtered for the expected Spt4/5 occupancy decay downstream of termination sites as well as the absence of TFB peaks to ensure the absence of active promoters and potentially promoter-proximal aCPSF1 peaks in the plotted region. TFB occupancy is depicted as control. The corresponding RNA-seq data for the plus strand are depicted on the right.

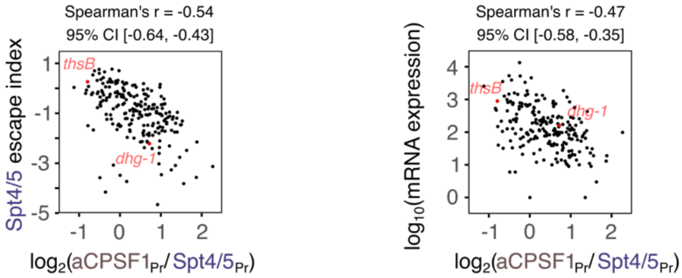

Supplementary Figure 9: aCPSF1 load on Spt4/5-containing TECs is negatively correlated to TEC escape

The left panel shows a scatter plots depicting the negative correlation between Spt4/5 escape index (mean of two biological replicates) and the relative load of aCPSF1 on the Spt4/5-bound TEC calculated as aCPSF1$_{Pr}$ to Spt4/5$_{Pr}$ ratio (geometric mean of two biological replicates, n=212 TUs). The right panel shows the correlation of aCPSF1 load calculated relative to Spt4/5$_{Pr}$ replicates (geometric mean of two biological replicates, n=211 TUs) with mRNA expression levels. Rockhopper [1] estimates of mRNA levels are based on two biological replicates.

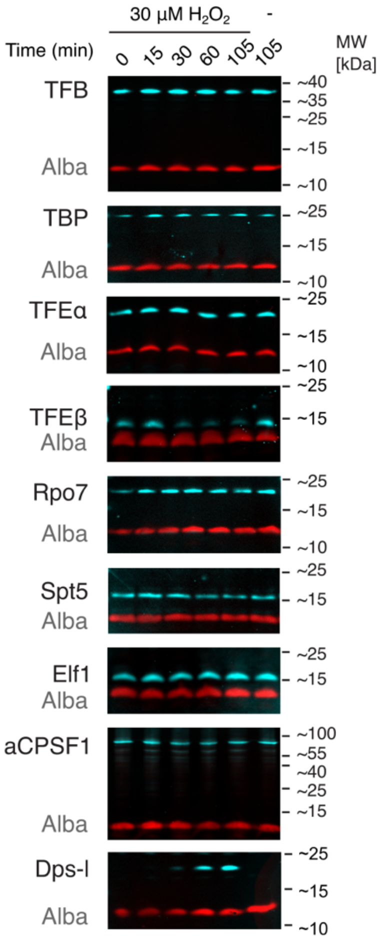

Supplementary Figure 10: TFEβ depletion in response to oxidative stress

Multiplex immunodetection of initiation factors (TFB, TBP, TFEα, TFEβ) RNAP subunits Rpo4/7, elongation factors (Spt4/5, ELf1) and termination factor aCPSF1 during a time course after H_2O_2-treatment (cyan signal). Detection of the oxidative stress marker protein Dps-l is shown as control. The stably expressed chromatin Alba (red signal) was used as loading control. A representative blot from three biological replicates is shown.

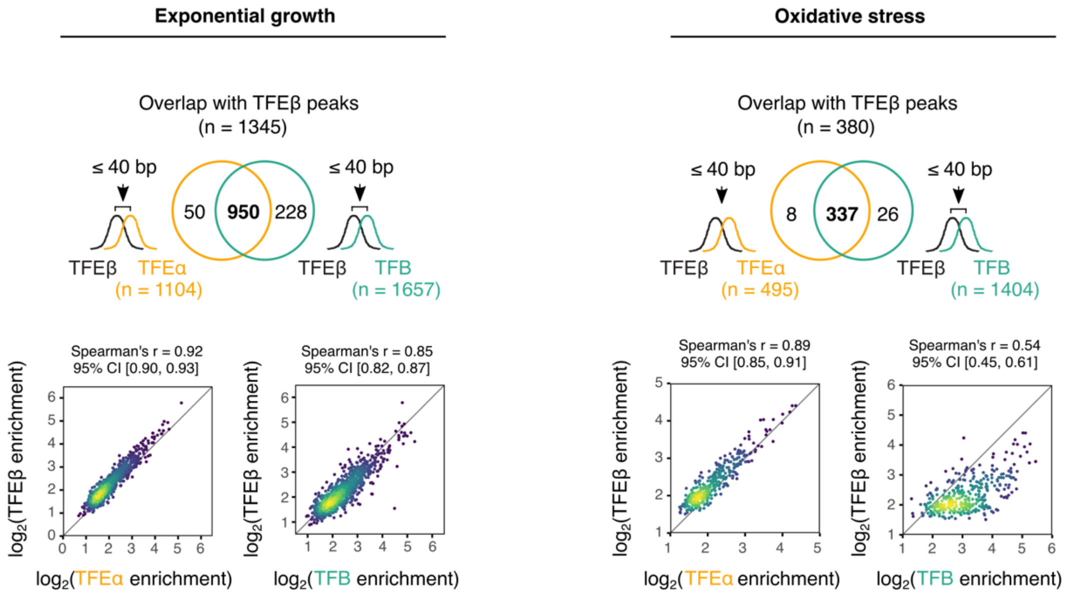

Supplementary Figure 11: Attenuated recruitment of TFE to PICs under oxidative stress conditions

The upper panels depict Venn diagrams of TFEα and TFB peaks that overlap with TFEβ peaks (as defined by maximum 40 bp distance between peaks) under exponential growth (left panel) and oxidative stress conditions (right panel). The total peak number for each factor is stated in brackets.

The lower panels depict scatter plots showing the genome-wide correlated enrichment for TFEβ peaks overlapping with both TFEα and TFB. Under oxidative stress conditions, TFEβ remain strongly correlated to TFEα and both factors show weaker enrichment compared to TFB. Data represent the geometric mean enrichment and arithmetic mean peak position of two biological replicates.

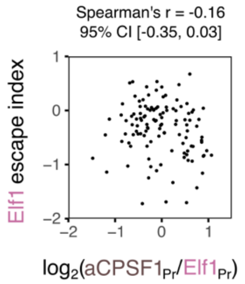

Supplementary Figure 12: aCPSF1 load on the TEC shows a weaker link to TEC escape under oxidative stress conditions

Scatter plot depicting the relation between the Elf1 escape index (mean of two biological replicates) and aCPSF1-load calculated as $aCPSF1_{Pr}$ to $Elf1_{Pr}$ ratio (geometric mean of two biological replicates, n=118 TUs). No significant correlation was detected.

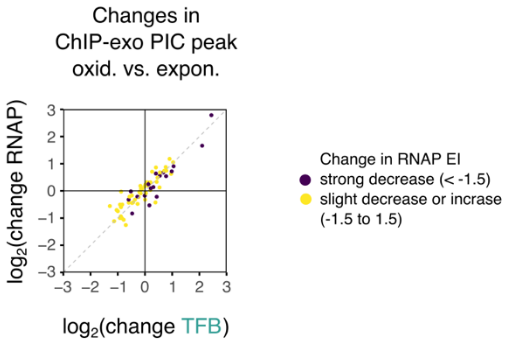

Supplementary Figure 13: TFB and RNAP PIC occupancy shows equal changes between growth conditions.

Scatter plot showing log_2-fold changes in ChIP-exo PIC signal between oxidative stress and exponential growth phase. PIC signal was calculated as ChIP-exo signal on the non-template strand over a window from -30 to +20 relative to TSS (Figure 1c). Promoters with both mapped TSS [2] and TSS prediction only based on start codon position and ChIP-exo profiles (see methods) were included (n=70). We centred the data by subtracting the mean value for all selected promoters. The promoters of TUs undergoing a larger reduction in RNAP escape index (< 1.5) under oxidative stress conditions compared to exponential growth are labelled in dark purple (n=19). All ChIP-exo quantifications were based on the geometric mean of three biological replicates.

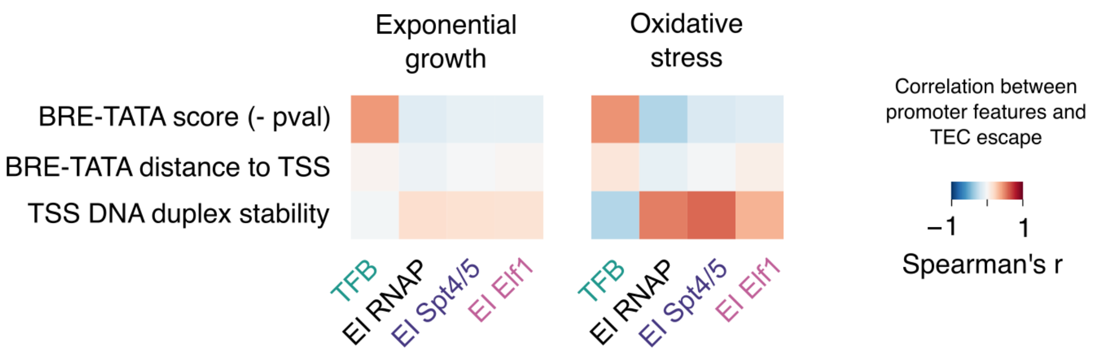

Supplementary Figure 14: Link between BRE-TATA core promoter elements with TEC escape

Heatmap depicting the correlation between the archaeal core promoter elements BRE-TATA and TEC escape calculated as escape indices for RNAP, Spt4/5 and Elf1 (mean of two biological replicates). 140 and 93 TUs were include for exponential growth and oxidative stress conditions, respectively. BRE-TATA elements were predicted by MEME for both sets of genes independently and the Spearman correlation was calculated between the motif strength for each promoter and the escape indices. Two controls are included: the positive correlation between TFB promoter occupancy and BRE-TATA score and the positive correlation of TSS DNA duplex stability with RNAP and Spt4/5 escape indices (see Figure 6).

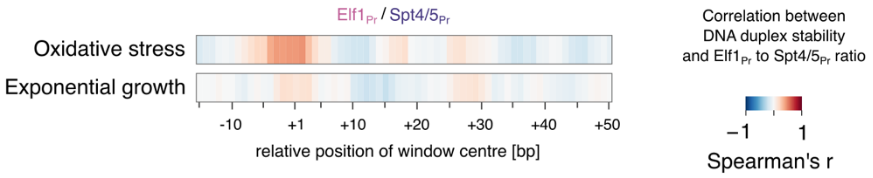

Supplementary Figure 15: Promoter-proximal TEC assembly is sensitive to DNA duplex stability around the TSS under oxidative stress.

Heatmaps depicting the correlation between DNA duplex stability and with the ratio of Elf1 to Spt4/5 occupancy in the promoter region (mean of two biological replicates) under oxidative stress (top) and exponential growth conditions (bottom). DNA duplex stability was calculated over a 7 bp sliding window for individual promoters (see methods). Selected TUs with mapped TSS were included (n=93 TUs for oxidative stress and n=140 TUs for exponential growth).

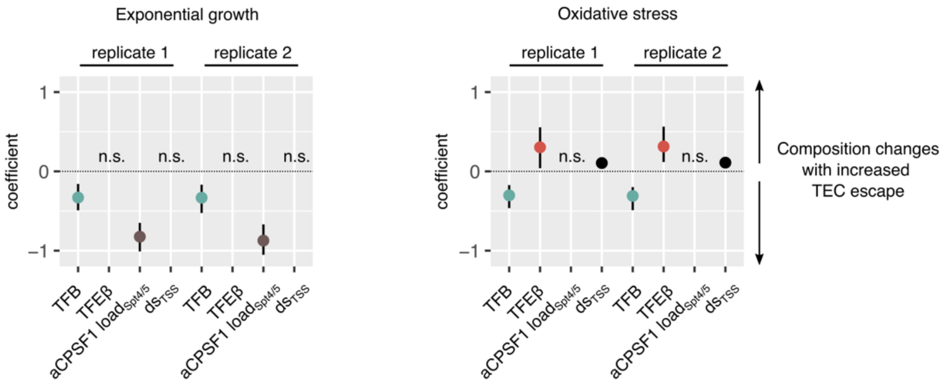

Likelihood-ratio χ^2-test

Explanatory variable	Exponential growth replicate 1	Exponential growth replicate 2	Oxidative stress replicate 1	Oxidative stress replicate 2
TFB	$< 10^{-3}$	$< 10^{-3}$	$< 10^{-3}$	$< 10^{-4}$
TFEβ	n.s.	n.s.	$< 10^{-2}$	$< 10^{-2}$
aCPSF1 load$_{Spt4/5}$	$< 10^{-14}$	$< 10^{-14}$	n.s.	n.s.
ds$_{TSS}$	n.s.	n.s.	$< 10^{-4}$	$< 10^{-5}$

Supplementary Figure 16: Multiple regression analysis of TEC escape under exponential growth and oxidative stress conditions

We generated negative binomial generalised linear models for TEC escape under exponential growth (left panel, n = 137 TUs) and oxidative stress conditions (right panel, n = 92 TUs) based on Spt4/5 escape indices (see methods). A set of four candidates for explanatory variables was tested: TFB and TFEβ promoter occupancy (log-transformed), aCPSF1 load on promoter-proximal TECs (log-transformed aCPSF1$_{Pr}$ to Spt4/5$_{Pr}$ ratio), and TSS DNA duplex stability. Explanatory variables were included in the final models if they improved the models significantly as determined by the likelihood ratio chi-squared test as depicted in the lower panel (threshold for inclusion of $p < 0.05$). “n.s.” marks explanatory variables not passing the likelihood ratio test for the model. Coefficient estimates for two biological replicates. Error bars represent bootstrapped 95% confidence intervals for the coefficients and for each replicate. Bootstrapping was used to validate the models. The models were consistent between the biological replicates.

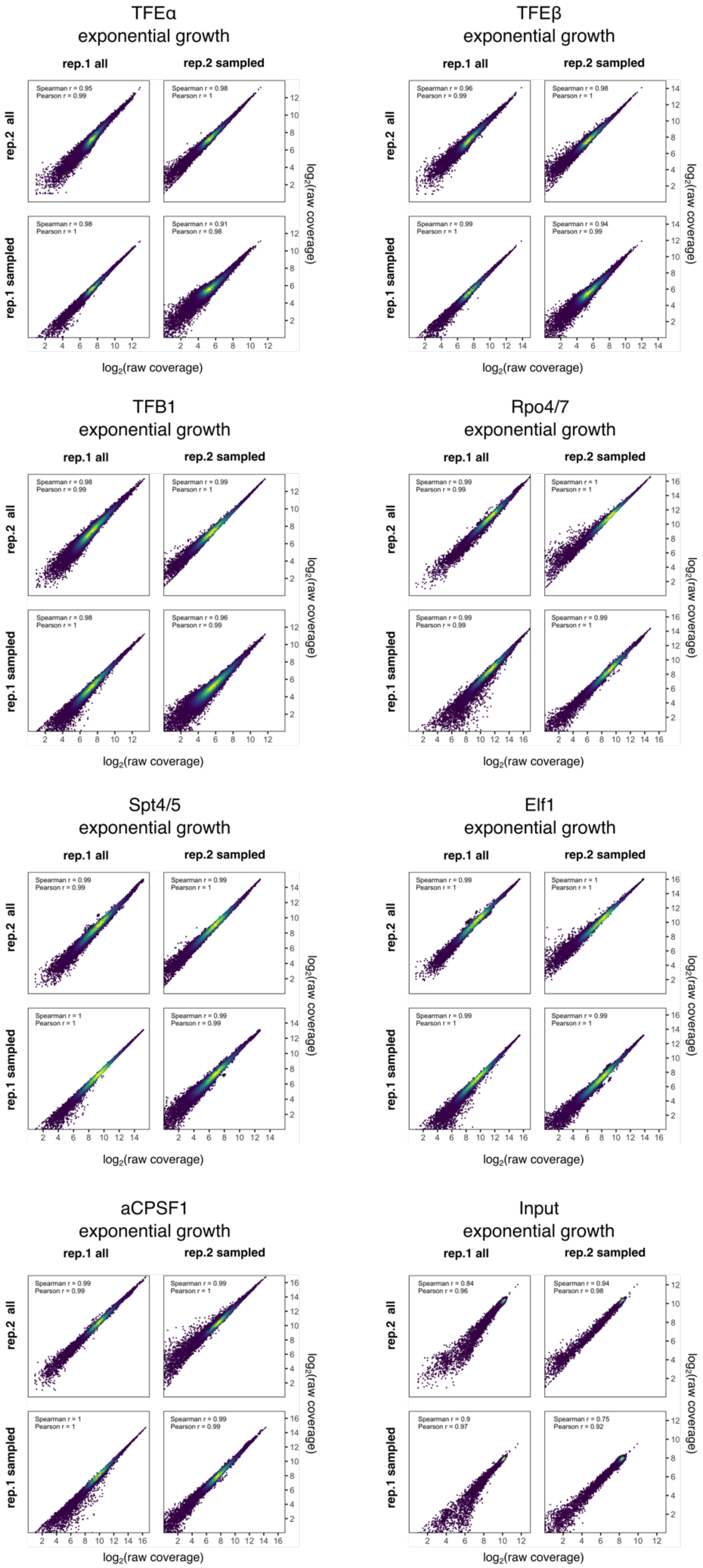
TFEα
exponential growth
TFEβ
exponential growth
TFB1
exponential growth
Rpo4/7
exponential growth
Spt4/5
exponential growth
Elf1
exponential growth
aCPSF1
exponential growth
Input
exponential growth
rep.1 all
rep.2 sampled
rep.2 all
rep.1 sampled
log2(raw coverage)

Supplementary Figure 17: Reproducibility of ChIP-seq samples and effect of read sampling for cells in the exponential growth phase

Heatscatter plots depicting the pairwise correlation of read coverage over consecutive 50 bp bins between two biological replicates (rep. 1 and rep. 2) for all ChIP-seq samples before and after sampling of reads to adjust read size distribution. All bins with minimum coverage of 1 were included.

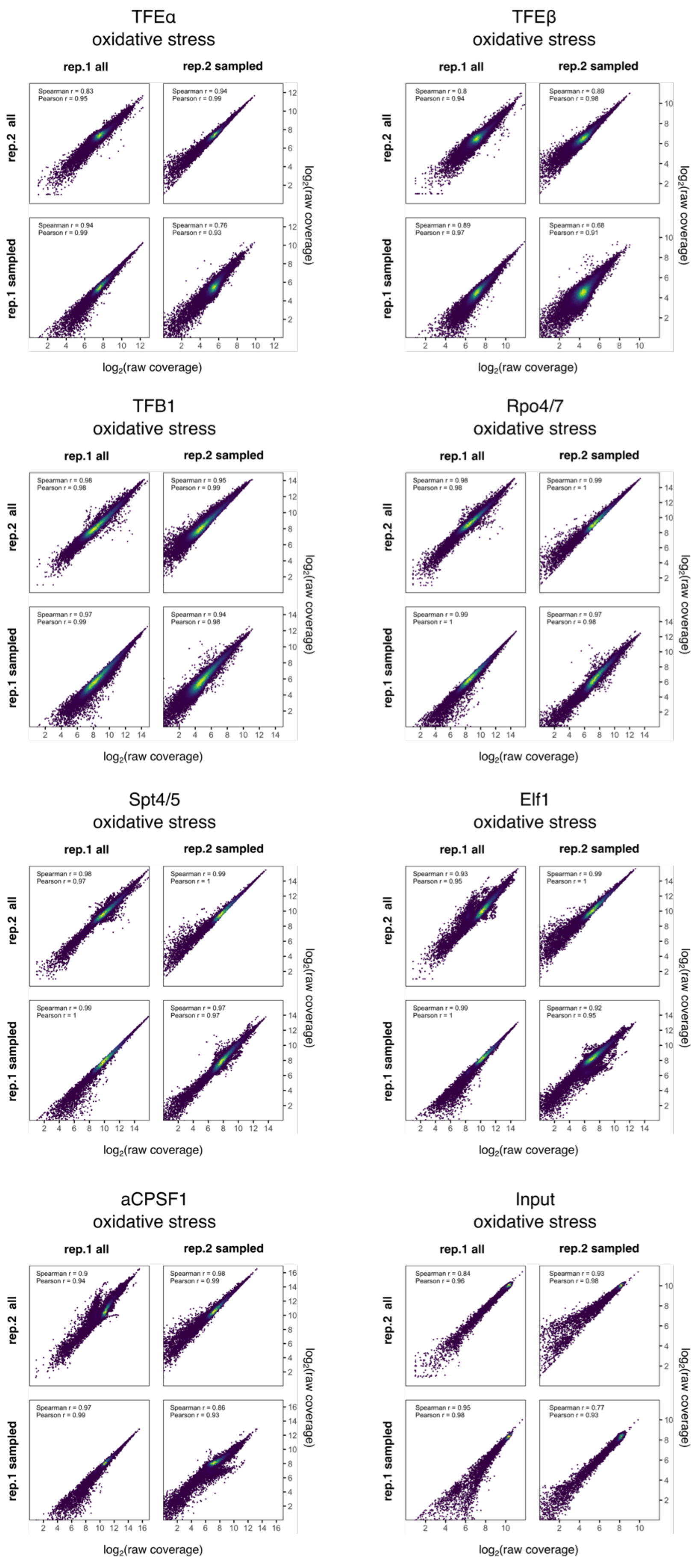
TFEα
oxidative stress
TFEβ
oxidative stress
TFB1
oxidative stress
Rpo4/7
oxidative stress
Spt4/5
oxidative stress
Elf1
oxidative stress
aCPSF1
oxidative stress
Input
oxidative stress
rep.1 all
rep.2 sampled
rep.2 all
rep.1 sampled
log2(raw coverage)

Supplementary Figure 18: Reproducibility of ChIP-seq samples and effect of read sampling for cells during oxidative stress

Heatscatter plots depicting the pairwise correlation of read coverage over consecutive 50 bp bins between two biological replicates (rep. 1 and rep. 2) for all ChIP-seq samples before and after sampling of reads to adjust read size distribution. All bins with minimum coverage of 1 were included.

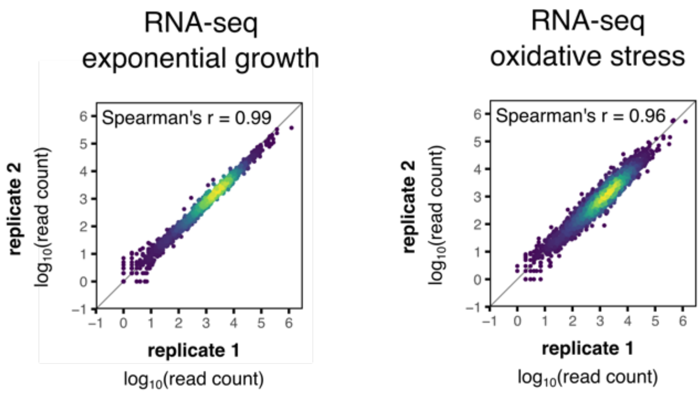

Supplementary Figure 19: Reproducibility of RNA-seq data

Raw read counts for all coding genes (n=3057) were correlated between biological replicates.

Supplementary References

1 Tjaden, B. De novo assembly of bacterial transcriptomes from RNA-seq data. *Genome Biology* **16**, 1, doi:10.1186/s13059-014-0572-2 (2015).

2 Wurtzel, O. *et al.* A single-base resolution map of an archaeal transcriptome. *Genome Res* **20**, 133-141, doi:10.1101/gr.100396.109 (2010).
