# Supplementary material for: Promoter-proximal elongation regulates transcription in archaea
Source: Nat Commun. 2021 Sep 17;12:5524. doi: 10.1038/s41467-021-25669-2 (PMC8448881; doi:10.1038/s41467-021-25669-2)
Supplement: Supplementary file 3 — Reporting Summary [file 41467_2021_25669_MOESM3_ESM.pdf]

nature research

Corresponding author(s): Fabian Blombach and Finn Werner

Last updated by author(s): 10-08-2020

Reporting Summary

Nature Research wishes to improve the reproducibility of the work that we publish. This form provides structure for consistency and transparency in reporting. For further information on Nature Research policies, see our Editorial Policies and the Editorial Policy Checklist.

Statistics

For all statistical analyses, confirm that the following items are present in the figure legend, table legend, main text, or Methods section.

n/a	Confirmed	
☐	☒	The exact sample size (*n*) for each experimental group/condition, given as a discrete number and unit of measurement
☐	☒	A statement on whether measurements were taken from distinct samples or whether the same sample was measured repeatedly
☐	☒	The statistical test(s) used AND whether they are one- or two-sided<br>*Only common tests should be described solely by name; describe more complex techniques in the Methods section.*
☐	☒	A description of all covariates tested
☐	☒	A description of any assumptions or corrections, such as tests of normality and adjustment for multiple comparisons
☐	☒	A full description of the statistical parameters including central tendency (e.g. means) or other basic estimates (e.g. regression coefficient) AND variation (e.g. standard deviation) or associated estimates of uncertainty (e.g. confidence intervals)
☐	☒	For null hypothesis testing, the test statistic (e.g. *F*, *t*, *r*) with confidence intervals, effect sizes, degrees of freedom and *P* value noted<br>*Give P values as exact values whenever suitable.*
☒	☐	For Bayesian analysis, information on the choice of priors and Markov chain Monte Carlo settings
☒	☐	For hierarchical and complex designs, identification of the appropriate level for tests and full reporting of outcomes
☐	☒	Estimates of effect sizes (e.g. Cohen's *d*, Pearson's *r*), indicating how they were calculated

Our web collection on statistics for biologists contains articles on many of the points above.

Software and code

Policy information about availability of computer code

Data collection	No software was used for data collection.
Data analysis	Deep sequencing data analysis:<br>bedtools v2.29.2; samtools 1.10; deeptools 3.4.3; meme 4.11.2; bowtie1.2.3; cutadapt 2.9; macs2 2.1.1; MACE 1.2; Rockhopper 2.03<br>R 3.6.2 with the following packages: Biostrings 2.54.0; GenomicRanges 1.38.0; ggplot2 3.3.2; idr 1.2; Rsamtools 2.2.3; rtracklayer 1.46.0; RVAideMemoire 0.9-77<br><br>Custom code for deep sequencing data analysis:<br>https://doi.org/10.5281/zenodo.5196117<br><br>Image analysis: ImageQuant TL

For manuscripts utilizing custom algorithms or software that are central to the research but not yet described in published literature, software must be made available to editors and reviewers. We strongly encourage code deposition in a community repository (e.g. GitHub). See the Nature Research guidelines for submitting code & software for further information.

Data

Policy information about availability of data

All manuscripts must include a data availability statement. This statement should provide the following information, where applicable:

- Accession codes, unique identifiers, or web links for publicly available datasets
- A list of figures that have associated raw data
- A description of any restrictions on data availability

All sequencing data files (ChIP-seq, ChIP-exo, permanganate ChIP-seq, RNA-seq, Cappable-seq) and the processed data were deposited at NCBI GEO under accession code GSE141290. The genome sequence of Saccharolobus solfataricus P2 has been deposited previously at NCBI under accession NC_002754.1.

Field-specific reporting

Please select the one below that is the best fit for your research. If you are not sure, read the appropriate sections before making your selection.

- [x] Life sciences
- [ ] Behavioural & social sciences
- [ ] Ecological, evolutionary & environmental sciences

For a reference copy of the document with all sections, see nature.com/documents/nr-reporting-summary-flat.pdf

Life sciences study design

All studies must disclose on these points even when the disclosure is negative.

Sample size	Sample size (as number of biological replicates) is two to three biological replicates as common in ChIP-seq experiments. No explicit power analysis was performed.
Data exclusions	Criteria for inclusion or exclusion of transcription units for the analysis of ChIP-seq, ChIP-exo, permanganate ChIP-seq, RNA-seq and Cappable-seq data are listed in the Material & Methods section. In brief we filtered for mappability, overlap of ChIP-seq signal between neighboring transcription units, and the absence of TU-internal promoters (sense or antisense).
Replication	Biological replicates (two to three) were used throughout all experiments. All attempts at replication were successful. For the reproducibility of data, see the Methods section and the Supplemental Item.
Randomization	Randomization was not relevant to this study.
Blinding	Blinding was not relevant to this study.

Reporting for specific materials, systems and methods

We require information from authors about some types of materials, experimental systems and methods used in many studies. Here, indicate whether each material, system or method listed is relevant to your study. If you are not sure if a list item applies to your research, read the appropriate section before selecting a response.

Materials & experimental systems

n/a	Involved in the study	
[ ]	[x]	Antibodies
[x]	[ ]	Eukaryotic cell lines
[x]	[ ]	Palaeontology and archaeology
[x]	[ ]	Animals and other organisms
[x]	[ ]	Human research participants
[x]	[ ]	Clinical data
[x]	[ ]	Dual use research of concern

Methods

n/a	Involved in the study	
[ ]	[x]	ChIP-seq
[x]	[ ]	Flow cytometry
[x]	[ ]	MRI-based neuroimaging

Antibodies

Antibodies used	All primary antibodies were custom antibodies made against recombinant purified protein. For immuno-detection of proteins, two different secondary antibodies were used: donkey anti-rabbit IgG Dylight680 (A120-208D6, Lot A120-208D6-5, Bethyl Laboratories) and donkey anti-sheep IgG Alexa488 (A-11015, Lot 1716970, Thermo Fisher).
Validation	All primary antibodies were validated using Western blots (Figure S7 and https://doi.org/10.7554/eLife.08378).

ChIP-seq

Data deposition

- [x] Confirm that both raw and final processed data have been deposited in a public database such as GEO.
- [x] Confirm that you have deposited or provided access to graph files (e.g. BED files) for the called peaks.

Data access links<br>*May remain private before publication.*	We deposited all sequencing data have at NCBI GEO under superseries GSE141290. Data are accessible with reviewer token "idgtgskghfaxduz"
Files in database submission	Raw sequencing files and bigwig coverage tracks are available for all biological replicates with the exception of cappable sRNA-seq data where we deposited bam files with the mapped reads as processed data files
Genome browser session<br>(e.g. UCSC)	https://genome.ucsc.edu/cgi-bin/hgTracks?hubUrl=https://raw.githubusercontent.com/surykartka/Sso_UCSC/master/myHub/hub.txt&genome=hub_2355503_SsoP2&position=lastDbPos

Methodology

Replicates	All data are from two biological replicates. We have used averaged data for all pairwise comparisons to keep the mansuscript short. We can provide plots for individual replicate comparisons upon request. ChIP-seq experiments with different antibodies were conducted on the same biological replicates.
Sequencing depth	Each replicate for chromatin input was sequenced to minimum depth of sequencing coverage of 180 (for uniquely mapped reads), ChIP-seq for RNA polymerase and other factors (Elf1, Spt4/5, aCPSF1) to minimum depth of sequencing coverage of 100, initiation factor ChIP-seq (TFB, TFEbeta, TFEalpha) to to minimum depth of sequencing coverage of 20. For all ChIP-seq experiments reads were paired-end. Details on mapping statistics are in the supplementary item.
Antibodies	All antibodies used for ChIP-seq are rabbit antibodies custom produced at Davids Biotechnology (Germany) against recombinant purified proteins.
Peak calling parameters	Peaks were identified with MACS2 in BEDPE mode, q 0.01 and with the call-summit sub-function in order to identify overlapping peaks. MACS2 output provides summit coordinates and quality scores for each peak, but the coordinates for each enriched region are not split between the overlapping peaks. For this reason, we used the peak summit positions to merge peaks from replicates with 40 bp max distance which should correspond to more than 50% overlap between the peaks using BEDTools window function. For the consistency analysis of the peaks between replicates based on p-values 66, we set a global IDR threshold of 0.05 using the Cran IDR package in R.
Data quality	Data quality was assessed by checking reproducibility. Peak enrichment is not an appropriate criterion for general transcription factors (i.e. high numbers of peaks expected, partially overlapping) distributed on small genomes. Because we used paired-end sequencing data for ChIP-seq experiments, MACS2 peak calling did not rely on model building that requires usually a high number of peaks with strong enrichment. Bedtools fisher test confirmed that initiation factor peaks are associated with previously identified mRNA and antisense TSSs in a highly significant manner.
Software	Basic conversion from fastq to bigwig coverage tracks involved alignment with bowtie, conversion to bam files with samtools, read sampling with custom code in R (see github.com/fblombac h/ChIP-seq) and coverage track generation including input normalisation with deeptools bam
